# Area-Level Social Vulnerability and Severe COVID-19: A Case–Control Study Using Electronic Health Records from Multiple Health Systems in the Southeastern Pennsylvania Region

**DOI:** 10.1007/s11524-024-00876-6

**Published:** 2024-05-13

**Authors:** Pricila H. Mullachery, Usama Bilal, Ran Li, Leslie A. McClure

**Affiliations:** 1 Department of Health Services Administration and Policy, College of Public Health, Temple University, 1301 Cecil B. Moore Ave., Philadelphia, PA 19122 USA; 2 Urban Health Collaborative, Dornsife School of Public Health, Drexel University, 3600 Market St, Philadelphia, PA 19104 USA; 3 Department of Epidemiology and Biostatistics, Dornsife School of Public Health, Drexel University, 3215 Market St, Philadelphia, PA 19104 USA

**Keywords:** COVID-19, Neighborhoods, Case control

## Abstract

Knowledge about neighborhood characteristics that predict disease burden can be used to guide equity-based public health interventions or targeted social services. We used a case-control design to examine the association between area-level social vulnerability and severe COVID-19 using electronic health records (EHR) from a regional health information hub in the greater Philadelphia region. Severe COVID-19 cases (*n* = 15,464 unique patients) were defined as those with an inpatient admission and a diagnosis of COVID-19 in 2020. Controls (*n* = 78,600; 5:1 control-case ratio) were a random sample of individuals who did not have a COVID-19 diagnosis from the same geographic area. Retrospective data on comorbidities and demographic variables were extracted from EHR and linked to area-level social vulnerability index (SVI) data using ZIP codes. Models adjusted for different sets of covariates showed incidence rate ratios (IRR) ranging from 1.15 (95% CI, 1.13–1.17) in the model adjusted for individual-level age, sex, and marital status to 1.09 (95% CI, 1.08–1.11) in the fully adjusted model, which included individual-level comorbidities and race/ethnicity. The fully adjusted model indicates that a 10% higher area-level SVI was associated with a 9% higher risk of severe COVID-19. Individuals in neighborhoods with high social vulnerability were more likely to have severe COVID-19 after accounting for comorbidities and demographic characteristics. Our findings support initiatives incorporating neighborhood-level social determinants of health when planning interventions and allocating resources to mitigate epidemic respiratory diseases, including other coronavirus or influenza viruses.

**Supplementary Information:**

The online version contains supplementary material available at 10.1007/s11524-024-00876-6.

## Introduction

The COVID-19 pandemic has highlighted longstanding injustices in the distribution of societal resources and their consequences for health disparities [1–3]. Low-income communities and racially minoritized groups are disproportionally affected by the COVID-19 pandemic. Mechanisms underlying these inequities include higher risk of exposure among minoritized groups—due to disproportionate rates of incarceration [4], disproportionate participation in the essential workforce and in jobs with unsafe work conditions [5, 6]—, increased susceptibility to severe and more debilitating outcomes, and reduced access to protective measures and health care [2, 7–9]. Understanding modifiable factors that contribute to these inequities is critical to inform policies toward mitigating morbidity and mortality in future disease outbreaks.

The neighborhood environment is a social determinant of health; in particular, neighborhood socioeconomic disadvantage is a key driver of health inequities. Research about neighborhood effects on outcomes such as all-cause mortality, cardiovascular disease risk, and pregnancy and birth outcomes has demonstrated that neighborhood characteristics such as socioeconomic status and built environment are strongly associated with health and health disparities [10–14], and the COVID-19 pandemic was no exception. For example, research using neighborhood-level cumulative case and death counts found that disadvantaged neighborhoods in US cities had higher COVID-19 incidence and mortality and lower vaccination rates and access to testing than more socially advantaged neighborhoods [1–3, 15–19]. These studies included a variety of datasets and neighborhood disadvantage metrics, but many used exclusively neighborhood-level data and aggregate counts of cases, hospitalizations, deaths, vaccines, or testing sites without accounting for individual-level characteristics.

There are also a number of studies examining individual-level clinical risk factors for COVID-19 morbidity and mortality [20]. These studies take advantage of electronic health records (EHR) containing a large number of clinical, laboratory, and imaging variables. Although these studies are important to guide individual clinical care, they have limited use within a public health framework, which focuses on populations and communities. A small number of studies have combined EHR with other sources of data on indicators of the social determinants of health in search of a perspective that is more relevant to guide public health interventions. For example, data from Veterans Health Administration (VHA) were linked to county-level SDOH indicators showing that the risk of COVID-19 increased with increased in adverse county-level indicators, such as percentage of residents without a college degree and percentage of residents living in crowded housing, after adjusting for individual-level covariates [21]. Other VHA-based studies have examining disparities in COVID-19 risk, but VHA data are not likely generalizable to nonveterans [22, 23].

This study is unique in that it takes advantage of EHR containing clinical data on comorbidity diagnosis and sociodemographic data linked to neighborhood-level data and uses a robust study design (case–control) to test a hypothesis based on an explicit conceptual framework. We tested the hypothesis that area-level social vulnerability (measured by a composite index) is associated with the occurrence of severe COVID-19 in the Southeastern Pennsylvania region (SEPA). This region has approximately 4 million residents, including those in the city of Philadelphia and the surrounding counties of Montgomery, Bucks, Chester, and Delaware. COVID-19 cumulative incidence in the SEPA region ranged from five cases per 100 residences in Bucks County to seven cases per 100 residents in Philadelphia in early 2021 [19]. Residents of this region have also been historically affected by environmental exposures from sources such as manufacturing facilities, oil refineries, and major highways [24, 25]. Some of the most affected areas by environmental exposures tend to be more densely populated and have high rates of poverty [26, 27], suggesting an accumulation of harmful factors to health.

### Conceptual Framework

**Fig. 1:**
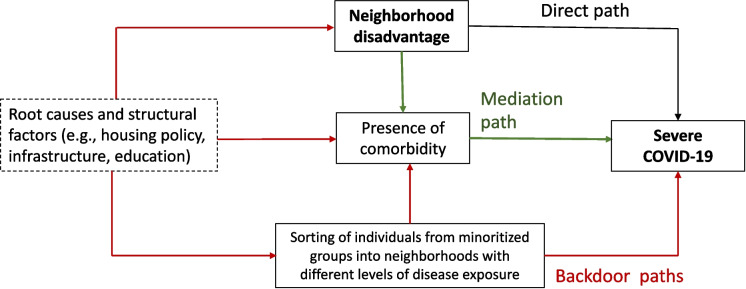
Conceptual framework. The dashed box outline represents historical policies that generated social stratification by race/ethnicity and for which the effects in health persist today. Green arrows represent the mediation path from neighborhood environment to comorbidities to COVID-19. Red arrows represent backdoor paths (i.e., confounding)

We used a neighborhood-centered approach to examine the association between area-level social vulnerability and COVID-19. A neighborhood-centered approach presents an alternative to biomedical and lifestyle models, which emphasize individual-level risk factors. In a neighborhood approach, individual-level factors are proximal to the health outcome(s) of interest and oftentimes on the pathway between neighborhood-level exposure and the outcome. In this context, a conceptual model demonstrating the expected pathways of association between variables at the individual- and neighborhood-level is critical to understand how adjustments for individual-level factors may change the associations between neighborhood-level factors and health outcomes. Figure 1 represents the conceptual model for the associations being tested in this study. In this figure, individual-level variables are mediators in the pathway between neighborhood social stratification and COVID-19 outcomes because neighborhood disadvantage is associated with chronic conditions (e.g., cardiovascular disease and diabetes) [10, 13, 28], and presence of comorbidities such as cardiovascular disease and diabetes is associated with COVID-19 outcomes and increase the risk of mortality [7, 29, 30]. At the same time, these comorbidities can also be confounders due to backdoor associations (Fig. 1) between neighborhood disadvantage and COVID-19 outcomes. For example, racially minoritized groups are more likely to (1) have higher prevalence of comorbidities, (2) live in disadvantaged neighborhoods due to historical policies that created segregated neighborhoods [31, 32], and (3) have higher rates of COVID-19 due to other exposures such as occupation.

## Methods

### Data Source and Study Setting

We used electronic health records (EHR) from HealthShare Exchange (HSX), a health information hub covering most hospitals in the Southeastern Pennsylvania Region (Philadelphia, Bucks, Delaware, Chester, and Montgomery counties) in addition to ambulatory care settings, long-term care, and community health settings, and patients covered by various insurance providers, Medicaid, and Medicare. We used data from HSX clinical data repository, which includes sociodemographic data (e.g., gender, age, race, ZIP code, marital status), and clinical data on hospital inpatient visits, emergency department encounters, diagnoses, and procedures. We processed approximately 3 million health care encounters.

### Study Design

This is a case–control retrospective study. Specifically, this study can be classified as a case cohort, where all cases of severe COVID-19 were identified, and a random sample from all members of the base population was selected to construct the control group [33]. To construct the sample, we first identified all individuals with a diagnosis of SARS-CoV-2 between March 1 and December 31 of 2020. Among those, we identified 15,464 unique cases of severe COVID-19, defined as those requiring inpatient care. Second, we selected 78,600 controls, a ratio of approximately five controls to one case. Controls were selected randomly from the source population after excluding all cases. To select a random sample of controls we assigned each patient in the clinical data repository a unique number using a random number generator, then ordered the numbers and selected the first 78,600 patients. Controls lived in one of the 220 neighborhoods (ZIP code areas) in five counties (i.e., Philadelphia, Bucks, Delaware, Chester, and Montgomery) with high coverage in the HSX clinical data repository. Controls were required to have at least one health care encounter in 2020 and one encounter in 2018–2019. These criteria increase the likelihood that the individual was residing in the area during the first year of the COVID-19 pandemic and had retrospective data (2018–2019) used to construct measures of exposure and covariates. Supplementary Fig. 1 shows the map of the region and compares the distribution of the population according to Census data and the distribution of controls in the analytical sample.

### Outcome

The outcome of the study was severe COVID-19, or inpatient cases, defined as those with at least one inpatient admission and a SARS-CoV-2 diagnosis (ICD-10 codes: B34.2, B97.29, U07.1) between March 1, 2020 to December 31, 2020. We included all cases that fit into the definition of outcome adopted.

### Exposure

Area-level social vulnerability was measured using the CDC’s social vulnerability index (SVI), defined in terms of community characteristics that affects their capacity to anticipate or recover from a disaster. This composite measure uses 15 variables related to four components: socioeconomic status, household composition and disability, monitory status and language, and housing type and transportation [34]. Measures such as the SVI have the advantage of capturing multiple dimensions of social and economic disadvantage [35]. The SVI has been used to characterize variations in COVID-19 outcomes and to allocate resources such as COVID-19 vaccines to communities with high need [36, 37]. We used 2015–2019 American Community Survey data 5-year estimates to calculate the SVI at level of ZIP code tabulation areas (ZCTAs). To calculate the SVI, the ZCTAs were ranked according to each of the variables in descending order, except for per capita income, which was ranked in ascending order, following Flanagan et al. [34]. After ranking the ZCTAs, we (1) calculated the percentile ranking for each variable, (2) calculated of the percentile ranking for the specific domains as the sum of the percentile ranks for each variable in the domain, and (3) calculated the overall SVI as the sum of the percentile ranks of the four domains.

Individual-level data from EHR were linked to area-level SVI using the ZIP code recorded in the most recent health care encounter prior to 2020 and mapping it to its corresponding ZCTA. For the analysis, the SVI, which originally ranges from 0 to 1, was multiplied by 10 to facilitate the interpretation of the results; thus, a change in one unit of the new measure can be interpreted as a 10% change in SVI.

### Covariates

We extracted data on diagnosis codes for known COVID-19 comorbidities (hypertension, diabetes, heart disease, renal disease, liver disease, cancer, and immunocompromised state) retrospectively from multiple healthcare encounters recorded between 2018 and 2019 (see Supplemental Box 1 for ICD-10 codes). We also extracted demographic variables, including age, sex, and race/ethnicity categorized into Hispanic, non-Hispanic American Indian and Alaska Native, non-Hispanic Asian and Pacific Islander, non-Hispanic Black, non-Hispanic White, and other race as recorded in the patient health record. Among all individuals included in the study, 12.8% had missing data for one or more comorbidities. In addition, 3.0% and 5.3% had missing data for race/ethnicity and marital status, respectively.

### Analytical Strategy

Cases and controls were characterized by demographic variables and according to the presence of comorbidities. We also created density plots showing the SVI distribution among cases and controls by race and ethnicity groups. We then constructed three primary models. The first model included the main exposure (SVI) and adjustments for sex and age. This model measures the association between social vulnerability and inpatient cases without adjusting for potential mediators or confounders, other than age or sex. The second model included comorbidities because they can act as confounders in the association between SVI and inpatient case (Fig. 1). The third model added the variable race/ethnicity. Adding race/ethnicity in the third model does not imply a biological difference among racial and ethnic groups. Rather, it reflects the fact that racially minoritized groups have been historically disadvantaged by discriminatory policies that impacted their residential distribution in the urban space and the resources made available in neighborhoods with large share of minoritized groups (Fig. 1).

We constructed models using the overall SVI and each of its four components: socioeconomic status, household composition and disability, monitory status and language, and housing type and transportation. We used mixed effects Poisson models with individuals nested within neighborhoods, based on the current ZIP code of residence (random intercept). We used Poisson regression rather than logistic regression and reported the incidence rate ratio (IRR) rather than the odds ratio to prevent overestimation of the association on the risk scale.

Among cases, 12.6% (1950 cases) had missing ZIP code values in the pre-pandemic period (2018–2019). For these cases, we used the ZIP code from their 2020 inpatient encounter. We conducted a sensitivity analysis excluding these cases to minimize potential bias from unmeasured residential mobility during the pandemic. We also constructed models stratified by different phases of the COVID-19 pandemic: (1) early pandemic (March 1 to May 31), summer (June 1 to July 31), fall (August 1 to October 31), and winter (November 1 to December 31) to assess changes related to COVID-19 surges and its impact on health system capacity. Finally, we used multiple imputation based on Markov Chain Monte Carlo augmentation to impute missing values for demographic characteristics. Analyses were conducted using STATA 17.

### Research Ethics Approval

This project was determined to be not human subject research and exempt from IRB review by the Drexel University Institutional Review Board, protocol #2,110,008,842.

## Results

Table 1 shows the sample characteristics. Among controls, mean age was 53 years (SD = 19), 63% were female, and 48% were married or had a partnered, while 43% were not married/partnered. Non-Hispanic white individuals were the majority among controls (57%); non-Hispanic Black, Hispanic, non-Hispanic Asian Pacific Islander, and American Indian and Alaska Native individuals were 23%, 6%, 4%, and 5% of the sample of controls, respectively. Among controls, prevalence of chronic conditions varied from 24% for hypertension to 2% for liver disease. Among COVID-19 inpatient cases, mean age was higher than that for controls, 65 years (SD = 18), 52% were female, 36% were married or had a partner, and 57% were not married/partnered. The distribution of inpatient cases by race/ethnicity was also different; non-Hispanic Black individuals were the largest group (42%) followed by non-Hispanic white, and Hispanic individuals representing 37% and 11% of the cases, respectively. The prevalence of chronic conditions was considerably higher among cases than controls, varying from 67% prevalence for hypertension to 6% for liver disease. Prevalence of hypertension, diabetes, and heart disease was about three times higher among cases vs. controls, and immunocompromised state was about seven times higher among cases than controls.

**Table 1:** Sample characteristics

	Total (*n* = 94,054)	Controls (*n* = 78,600)	Cases (*n* = 15,464)
Mean (age/SD)	55 (20)	53 (19)	65 (18)
Female (%)	61.4	63.3	51.9
Marital status			
Married/partnered (%)	46.5	48.4	36.4
Not married/partnered (%)	46.0	43.8	57.5
Undefined (%)	7.5	7.8	6.1
Race/ethnicity			
Hispanic or Latino(a) (%)	6.8	6.0	11.2
American Indian and Alaska Native (%)	4.4	5.3	0.1
Asian and Pacific Islander (%)	3.7	3.7	3.5
Black (%)	26.0	22.9	41.7
White (%)	53.7	57.1	36.6
Other (%)	5.3	5.0	6.9
Diagnosis of chronic conditions (2018–2019)			
Hypertension (%)	32.4	24.6	66.5
Diabetes (%)	15.6	10.4	38.2
Heart disease (%)	15.7	11.2	35.6
Liver disease (%)	2.9	2.1	6.4
Renal disease (%)	7.9	4.2	24.0
Cancer (%)	7.9	7.3	10.9
Immunocompromised state (%)	6.4	3.1	20.7

**Fig. 2:**
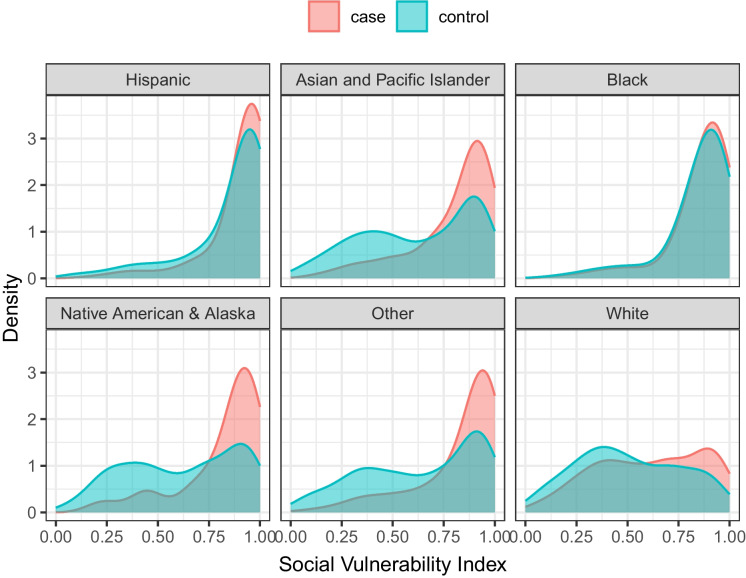
Distribution of cases and controls by neighborhood social vulnerability index stratified by race/ethnicity groups

Figure 2 shows the distribution of cases and controls over the SVI by race/ethnicity groups. Racially minoritized groups are concentrated in neighborhoods with high SVI. Non-Hispanic white individuals are distributed more evenly across the SVI variable. In general, cases were more concentrated in high-SVI neighborhoods compared to controls.

Table 2 shows the main results from models that included the overall SVI and models that included only one of the SVI components. For the overall SVI, models adjusted for different sets of covariates showed incidence rate ratios (IRR) ranging from 1.15 (95% CI, 1.13–1.17) in the model adjusted for individual-level age, sex, and marital status to 1.09 (95% CI, 1.08–1.11) in the fully adjusted model, which included individual-level comorbidities and race/ethnicity. Thus, the fully adjusted model indicates that a 10% higher area-level SVI was associated with a 9% higher risk of severe COVID-19. Secondary analyses excluding individuals who did not have ZIP code data prior to 2020 (Model 3.1) and model with imputed missing values (Model 3.2) showed similar results. Overall, models including only one of the SVI components (socioeconomic status, household composition, minority status and language, and housing type and transportation) showed weaker associations, but coefficients from models using the socioeconomic status component (IRR = 1.13–1.08, across models 1 to 3) were generally similar to coefficients from models using the overall SVI.

**Table 2:** Incidence rate ratio (IRR) and 95% CI for the association between retrospective measures of area-level social vulnerability and severe COVID-19, March to December 2020

	Model 1		Model 2		Model 3		Model 3.1		Model 3.2	
Models	IRR	95% CI	IRR	95% CI	IRR	95% CI	IRR	95% CI	IRR	95% CI
SVI overall	1.15	1.13–1.17	1.12	1.10–1.13	1.09	1.08–1.11	1.10	1.08–1.11	1.08	1.07–1.10
SVI socioeconomic status	1.13	1.11–1.14	1.09	1.08–1.11	1.08	1.06–1.09	1.08	1.06–1.09	1.07	1.05–1.08
SVI household composition	1.08	1.07–1.10	1.07	1.05–1.08	1.05	1.04–1.07	1.06	1.04–1.07	1.05	1.03–1.06
SVI minority status and language	1.11	1.09–1.13	1.09	1.07–1.11	1.07	1.06–1.09	1.07	1.06–1.09	1.06	1.05–1.08
SVI housing type and transportation	1.07	1.05–1.09	1.06	1.04–1.08	1.05	1.04–1.07	1.05	1.04–1.07	1.05	1.03–1.06

Model 1: model adjusted for age, sex, marital status and excluding records missing data on comorbidities (*n* = 77,396)

Model 2: model adjusted for age, sex, marital status, comorbidities) (*n* = 77,396)

Model 3: model adjusted for age, sex, marital status, comorbidities, and race/ethnicity) (*n* = 75,187)

Model 3.1: model 3 excluding inpatient cases without an encounter prior to the pandemic (*n* = 73,468)

Model 3.2: model 3 with multiple imputation of missing values (*n* = 94,096)

**Fig. 3:**
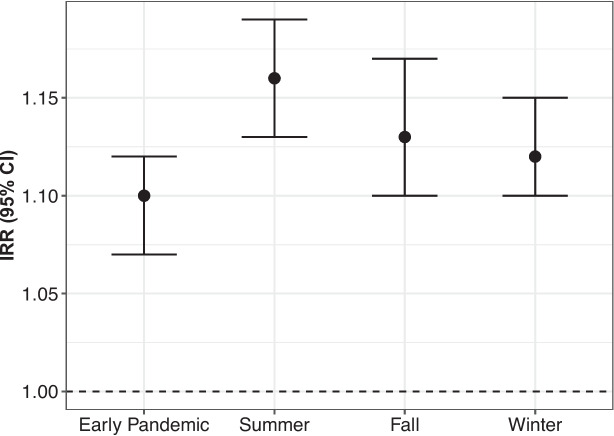
Incidence rate ratio (IRR) and 95% CI for the fully adjusted model stratified by periods in 2020. Early pandemic: from March 1 to May 31; summer: from June 1 to July 31; fall: from August 1 to October 31; and winter: from November 1 to December 31

Finally, Fig. 3 shows the IRR and 95% confidence intervals for the fully adjusted model (Model 3) stratified by different periods during the pandemic in 2020. Stratified models showed slight variation in the magnitude of the association between SVI and COVID-19 risk, but overall, the association was robust throughout the year.

## Discussion

In this case–control study using EHR from multiple healthcare systems and insurers in the SEPA region, we found a strong and persistent association between area-level social vulnerability and risk of severe COVID-19, defined as a case that required an inpatient visit. The association persisted even after adjusting for a number of comorbidities ascertained by retrospective diagnosis from EHR. A 10% higher SVI was associated with a 9% to 15% higher risk of COVID-19, depending on the set of covariates adjusted.

The patterns identified are consistent with the hypothesis that areas with high vulnerability do not just concentrate populations with high burden of disease, but that the conditions in which people live in these neighborhoods are associated with higher burden of disease. In this study, inpatient cases of COVID-19 were more likely to be from high-vulnerability neighborhoods, even after accounting for several comorbidities associated with COVID-19 and a number of demographic factors. In addition, given the fact that we used social vulnerability measures from retrospective data and SARS-CoV-2 was a new virus in 2020, reverse causation is not possible in this case.

The multiple sets of adjustments guided by the conceptual framework cover different possible association pathways between area-level vulnerability and COVID-19. The model with fewer adjustments shows a stronger association, likely due to the fact that these high-vulnerability neighborhoods also tend to concentrate sicker/more susceptible individuals, which is a source of confounding. The data also shows that high-vulnerability neighborhoods tend to concentrate individuals from racially minoritized groups who are likely exposed to other structural determinants of health [38, 39]. However, even after adjusting for several factors, the association persisted, indicating that social vulnerability is also directly associated with COVID-19 regardless of the presence of comorbidity and sociodemographic factors measured at the individual level. However, since comorbidities can also function as a mediator in the pathway between social vulnerability and COVID-19, adjusted models may be underestimating the main association. In this context, it is reasonable to believe that the true association is likely between that shown in models 1 (minimal adjustment excluding potential mediators) and model 3 (fully adjusted).

We found generally consistent results when examining different components of the SVI. Exploring different SVI components is particularly helpful to rule out issues related to the inclusion of variables such as race/ethnicity, which is not itself a marker of socioeconomic deprivation, but is linked to deprivation via racist policies [35]. Models including only the socioeconomic component of the SVI—which includes the variables percent of the residents living below poverty, percent unemployment, percent without a high school diploma, and average income, but does not include race or age composition—point to the same overall findings. Exploring specific SVI components also helps to rule out potential issues related to the inclusion of race and age both as individual-level variables and as neighborhood-level metrics in two of the SVI components [35].

Lastly, models stratified by periods of the pandemic showed consistent results. In the early pandemic period, the magnitude of the association between social vulnerability and severe COVID-19 risk was smaller compared to the subsequent periods. This could be due to overall low rates of hospitalizations among people from socially vulnerable neighborhoods who were more likely to face barriers to care and potentially dying at home [40] due to limited hospital capacity. This issue may have led to an underestimation of inpatient cases from socially vulnerable neighborhoods, leading to an underestimation of the risk ratio during this period.

Our findings are consistent with the literature examining the association between area-level socioeconomic measures and COVID-19 risk. For example, despite the differences between veteran and nonveteran populations [41], studies using EHR from Veteran Affairs (VA) linked to county-level social determinants of health [21], and neighborhood-level social vulnerability using the SVI and other composite metrics [23], showed an increased risk of COVID-19 infection and an increased risk of hospitalization in areas with greater socioeconomic adversity.

Our paper documents the association between neighborhood-level social disadvantage and the risk of severe COVID-19 in a population served by several healthcare systems in a large urban area. Our findings indicate that addressing area-level disadvantage is critical; by reducing social vulnerability, we may not just reduce the burden of COVID-19 and other potentially epidemic respiratory diseases directly, but we may also improve population health among those who need the most, which is critical to address health inequities. Information on these area-level characteristics can also guide equity-based public health interventions such as vaccination distribution or tailored social services to prevent hospitalizations and deaths in communities that concentrate disadvantage. In fact, this strategy was used by jurisdictions across the country to guide allocation of COVID-19 vaccines [42] with some showing promising results [43–45].

### Limitations

This study has multiple strengths, including a well-designed sample construction and analytical approach, a large sample size distributed across over 200 neighborhoods (ZCTAs), and use of the retrospective data to measure exposure and confounders. However, there are also has limitations. One limitation is the possibility of selection bias; the control group included individuals who had at least on visit in the previous year, so those who are less likely to seek health care, either because they are healthier or because of barriers to access are less likely to be included in the control group. For example, differences in health seeking behaviors between females and males are well documented and the female/male ratio in our control sample confirms the pattern that females are more likely to use health care. In addition, limiting individuals in the control group to those who had at least one encounter in 2020 may also have led to including sicker patients, particularly as health care utilization for non-urgent care declined in 2020 due to the pandemic [46, 47]. Considering these issues, a control group composed of sicker individuals (compared to the overall population) may lead to an underestimation of the true associations we would have found at the population level. On the other hand, comorbidities may be underreported in the EHR. Even though we used retrospective data to capture records of diagnosis in the 2018–2019 period, some conditions such as hypertension are slightly lower in the sample when compared to population survey data [48], which can be due to underreporting of these conditions. This is a misclassification problem, i.e., individuals with comorbidities such as hypertension are misclassified as not having hypertension. This misclassification results in incomplete control for confounding, which may bias the results towards the direction of the confounding [49]; in this case, the bias would be away from the null, based on the comparison of models 1 and 2. Future work using this data set should “look back” at additional years when creating retrospective measures of prevalence to examine whether the lower prevalence of certain chronic conditions is a result of a relatively healthier user population or inconsistent reporting of chronic conditions in the EHR.

## Conclusions

In this study, we found that individuals in neighborhoods with high social vulnerability were more likely to have severe COVID-19 after accounting for individual comorbidities and demographic characteristics. Our findings support initiatives that incorporate area-level social determinants of health when planning interventions and allocating resources to mitigate epidemic respiratory diseases, including other coronaviruses and influenza, which has historically shown similar disparities to COVID-19 [50]. By tailoring preventive measures to vulnerable communities, the number of cases requiring hospitalization could be reduced significantly, thus reducing the burden on this population and on the healthcare system in general. These results would likely extend to other diseases, making area-level interventions even more important to combat health inequities.

### Supplementary Information

Below is the link to the electronic supplementary material.

Supplementary file1 (DOCX 4012 KB)

## References

[1] Bilal U, Tabb LP, Barber S, Diez Roux AV. Spatial inequities in COVID-19 testing, positivity, confirmed cases, and mortality in 3 US cities: an ecological study. *Ann Intern Med*. 2021;174(7):936–44. 33780289 10.7326/M20-3936 PMC8029592

[2] Bilal U, Mullachery PH, Schnake-Mahl A, et al. Heterogeneity in spatial inequities in COVID-19 vaccination across 16 large US cities. *Am J Epidemiol*. 2022;191(9):1546–56. 35452081 10.1093/aje/kwac076 PMC9047229

[3] Mullachery PH, Li R, Melly S, et al. Inequities in spatial accessibility to COVID-19 testing in 30 large US cities. *Soc Sci Med*. 2022;310:115307. 36049353 10.1016/j.socscimed.2022.115307 PMC9420026

[4] LeMasters K, Brinkley-Rubinstein L, Maner M, Peterson M, Nowotny K, Bailey Z. Carceral epidemiology: mass incarceration and structural racism during the COVID-19 pandemic. *Lancet Public Health*. 2022;7(3):e287–90. 35247354 10.1016/S2468-2667(22)00005-6 PMC8890762

[5] Chen Y-H, Glymour M, Riley A, et al. Excess mortality associated with the COVID-19 pandemic among Californians 18–65 years of age, by occupational sector and occupation: march through November 2020. *PLoS ONE*. 2021;16(6):e0252454. 34086762 10.1371/journal.pone.0252454 PMC8177528

[6] Goldman N, Pebley AR, Lee K, Andrasfay T, Pratt B. Racial and ethnic differentials in COVID-19-related job exposures by occupational standing in the US. *PLoS ONE*. 2021;16(9):e0256085. 34469440 10.1371/journal.pone.0256085 PMC8409606

[7] Pranata R, Huang I, Lim MA, Wahjoepramono EJ, July J. Impact of cerebrovascular and cardiovascular diseases on mortality and severity of COVID-19–systematic review, meta-analysis, and meta-regression. *J Stroke Cerebrovasc Dis*. 2020;29(8):104949. 32410807 10.1016/j.jstrokecerebrovasdis.2020.104949 PMC7221373

[8] Romano SD, Blackstock AJ, Taylor EV, et al. Trends in racial and ethnic disparities in COVID-19 hospitalizations, by region—United States, March–December 2020. *Morb Mortal Wkly Rep*. 2021;70(15):560. 10.15585/mmwr.mm7015e2 PMC8344991 33857068

[9] Schnake-Mahl AS, Lazo M, Dureja K, Ehtesham N, Bilal U. Racial and ethnic inequities in occupational exposure across and between US cities. *SSM-Population Health*. 2021;16:100959. 34805478 10.1016/j.ssmph.2021.100959 PMC8590507

[10] Boylan JM, Robert SA. Neighborhood SES is particularly important to the cardiovascular health of low SES individuals. *Soc Sci Med*. 2017;188:60–8. 28732236 10.1016/j.socscimed.2017.07.005 PMC5563460

[11] Roux AVD, Borrell LN, Haan M, Jackson SA, Schultz R. Neighbourhood environments and mortality in an elderly cohort: results from the cardiovascular health study. *J Epidemiol Community Health*. 2004;58(11):917–23. 15483307 10.1136/jech.2003.019596 PMC1732601

[12] Roux AVD, Merkin SS, Arnett D, et al. Neighborhood of residence and incidence of coronary heart disease. *N Engl J Med*. 2001;345(2):99–106. 11450679 10.1056/NEJM200107123450205

[13] Roux AVD, Mujahid MS, Hirsch JA, Moore K, Moore LV. The impact of neighborhoods on CV risk. *Glob Heart*. 2016;11(3):353–63. 27741982 10.1016/j.gheart.2016.08.002 PMC5098701

[14] Metcalfe A, Lail P, Ghali WA, Sauve RS. The association between neighbourhoods and adverse birth outcomes: a systematic review and meta-analysis of multi-level studies. *Paediatr Perinat Epidemiol*. 2011;25(3):236–45. 21470263 10.1111/j.1365-3016.2011.01192.x

[15] Scannell Bryan M, Sun J, Jagai J, et al. Coronavirus disease 2019 (COVID-19) mortality and neighborhood characteristics in Chicago. *Ann Epidemiol*. 2021;56:47-54.e5. 33181262 10.1016/j.annepidem.2020.10.011 PMC7678719

[16] Sung B. A spatial analysis of the effect of neighborhood contexts on cumulative number of confirmed cases of COVID-19 in US Counties through October 20 2020. *Prev Med*. 2021;147:106457. 33607122 10.1016/j.ypmed.2021.106457 PMC7886632

[17] Samuels-Kalow ME, Dorner S, Cash RE, et al. Neighborhood disadvantage measures and COVID-19 cases in Boston, 2020. *Public Health Rep*. 2021;136(3):368–74. 33729070 10.1177/00333549211002837 PMC8580391

[18] Cm K, Oral E, Straif-Bourgeois S, Rung AL, Peters ES. The effect of area deprivation on COVID-19 risk in Louisiana. *PLoS One*. 2020;15(12):e0243028. 33270701 10.1371/journal.pone.0243028 PMC7714106

[19] Rizaldi AA, Xie S, Hubbard RA, Himes BE. Neighborhood characteristics and COVID-19 incidence and mortality in southeastern Pennsylvania. *AMIA Annu Symp Proc*. 2022;2022:422–31. PMC9285166 35854746

[20] Li J, Huang DQ, Zou B, et al. Epidemiology of COVID-19: a systematic review and meta-analysis of clinical characteristics, risk factors, and outcomes. *J Med Virol*. 2021;93(3):1449–58. 32790106 10.1002/jmv.26424 PMC7436673

[21] Abdel Magid HS, Ferguson JM, Van Cleve R, Purnell AL, Osborne TF. Differences in COVID-19 risk by race and county-level social determinants of health among veterans. *Int J Environ Res Public Health*. 2021;18(24):13140. 34948748 10.3390/ijerph182413140 PMC8701661

[22] Rentsch CT, Kidwai-Khan F, Tate JP, et al. Patterns of COVID-19 testing and mortality by race and ethnicity among United States veterans: a nationwide cohort study. *PLoS Med*. 2020;17(9):e1003379. 32960880 10.1371/journal.pmed.1003379 PMC7508372

[23] Wong MS, Brown AF, Washington DL. Inclusion of race and ethnicity with neighborhood socioeconomic deprivation when assessing COVID-19 hospitalization risk among California Veterans Health Administration Users. *JAMA Network Open*. 2023;6(3):e231471. 36867407 10.1001/jamanetworkopen.2023.1471 PMC9984969

[24] McKeon TP, Vachani A, Penning TM, Hwang W-T. Air pollution and lung cancer survival in Pennsylvania. *Lung Cancer*. 2022;170:65–73. 35716633 10.1016/j.lungcan.2022.06.004 PMC9732862

[25] Auchincloss A, De Roos AJ. RE: statement on the health effects of refineries and implications for the S Philadelphia refinery. Philadelphia, PA. 2019. https://www.phila.gov/media/20191204161537/Auchincloss-et-al-Statement-on-the-Health-Effectsof-Refineries-and-Implications-for-S-Phila-Refinery.pdf. Accessed 1 Jun 2023

[26] Klein NJ, Guerra E, Smart MJ. The Philadelphia story: age, race, gender and changing travel trends. *J Transp Geogr*. 2018;69:19–25. 10.1016/j.jtrangeo.2018.04.009

[27] Zhu Y, McKeon TP, Tam V, Vachani A, Penning TM, Hwang W-T. Geographic differences in lung cancer incidence: a study of a major metropolitan area within Southeastern Pennsylvania. *Int J Environ Res Public Health*. 2020;17(24):9498. 33352953 10.3390/ijerph17249498 PMC7767044

[28] Bilal U, Auchincloss AH, Diez-Roux AV. Neighborhood environments and diabetes risk and control. *Curr DiabRep*. 2018;18:1–10. 10.1007/s11892-018-1032-2 29995252

[29] Ssentongo P, Ssentongo AE, Heilbrunn ES, Ba DM, Chinchilli VM. Association of cardiovascular disease and 10 other pre-existing comorbidities with COVID-19 mortality: a systematic review and meta-analysis. *PLoS ONE*. 2020;15(8):e0238215. 32845926 10.1371/journal.pone.0238215 PMC7449476

[30] Silverio A, Di Maio M, Citro R, et al. Cardiovascular risk factors and mortality in hospitalized patients with COVID-19: systematic review and meta-analysis of 45 studies and 18,300 patients. *BMC Cardiovasc Disord*. 2021;21:1–13. 33413093 10.1186/s12872-020-01816-3 PMC7789083

[31] Riley AR. Neighborhood disadvantage, residential segregation, and beyond—lessons for studying structural racism and health. *J Racial Ethn Health Disparities*. 2018;5:357–65. 28573643 10.1007/s40615-017-0378-5

[32] Swope CB, Hernández D, Cushing LJ. The relationship of historical redlining with present-day neighborhood environmental and health outcomes: a scoping review and conceptual model. *J Urban Health*. 2022;99(6):959–83. 35915192 10.1007/s11524-022-00665-z PMC9342590

[33] Wacholder S, Silverman DT, McLaughlin JK, Mandel JS. Selection of controls in case-control studies: iII Design options. *Am J Epidemiol*. 1992;135(9):1042–50. 1595690 10.1093/oxfordjournals.aje.a116398

[34] Flanagan BE, Hallisey EJ, Adams E, Lavery A. Measuring community vulnerability to natural and anthropogenic hazards: the Centers for Disease Control and Prevention’s Social Vulnerability Index. *J Environ Health*. 2018;80(10):34. 32327766 PMC7179070

[35] Trinidad S, Brokamp C, Mor Huertas A, et al. Use of area-based socioeconomic deprivation indices: a scoping review and qualitative analysis: study examines socioeconomic deprivation indices. *Health Aff*. 2022;41(12):1804–11. 10.1377/hlthaff.2022.00482 36469826

[36] Barry V, Dasgupta S, Weller DL, et al. Patterns in COVID-19 vaccination coverage, by social vulnerability and urbanicity—United States, December 14, 2020–May 1, 2021. *Morb Mortal Wkly Rep*. 2021;70(22):818. 10.15585/mmwr.mm7022e1 PMC8174677 34081685

[37] De Ramos IP, Lazo M, Schnake-Mahl A, et al. COVID-19 outcomes among the hispanic population of 27 large US cities, 2020–2021. *Am J Public Health*. 2022;112(7):1034–44. 35588187 10.2105/AJPH.2022.306809 PMC9222469

[38] Crear-Perry J, Correa-de-Araujo R, Lewis Johnson T, McLemore MR, Neilson E, Wallace M. Social and structural determinants of health inequities in maternal health. *J Womens Health*. 2021;30(2):230–5. 10.1089/jwh.2020.8882 PMC8020519 33181043

[39] Darity WA Jr. Employment discrimination, segregation, and health. *Am J Public Health*. 2003;93(2):226–31. 12554574 10.2105/AJPH.93.2.226 PMC1447721

[40] COVID-19 mortality update — United States, 2022. *MMWR Morb Mortal Wkly Rep*. 2023;72:493–6. 10.15585/mmwr.mm7218a4 PMC10168601 37141157

[41] Betancourt JA, Granados PS, Pacheco GJ, et al. *Exploring health outcomes for US veterans compared to non-veterans from 2003 to 2019*. 2021: MDPI. p. 604. 10.3390/healthcare9050604 PMC8158130 34070037

[42] Tounkara MS. *How the social vulnerability index (SVI) provides insights into vaccination coverage inequities*. Center for Disease Control and Prevention Blog. 2023. https://blogs.cdc.gov/healthequity/2023/08/29/how-the-social-vulnerability-index-svi-provides-insights-into-vaccination-coverage-inequities/. Accessed 30 Oct 2023

[43] Assoumou SA, Peterson A, Ginman E, et al. Addressing inequities in SARS-CoV-2 vaccine uptake: the Boston Medical Center health system experience. *Ann Intern Med*. 2022;175(6):879–84. 35576586 10.7326/M22-0028

[44] Chen Y, Tao R, Downs J. Location optimization of COVID-19 vaccination sites: case in Hillsborough County, Florida. *Int J Environ Res Public Health*. 2022;19(19):12443. 36231743 10.3390/ijerph191912443 PMC9566030

[45] Bibbins-Domingo K, Petersen M, Havlir D. Taking vaccine to where the virus is—equity and effectiveness in coronavirus vaccinations. *Am Med Assoc*. 2021:e210213 10.1001/jamahealthforum.2021.0213 36218794

[46] Kruizinga MD, Peeters D, van Veen M, et al. The impact of lockdown on pediatric ED visits and hospital admissions during the COVID19 pandemic: a multicenter analysis and review of the literature. *Eur J Pediatr*. 2023;180(7):2271–2279 10.1007/s00431-021-04015-0 PMC7959585 33723971

[47] Barten DG, Latten GHP, van Osch FHM. Reduced emergency department utilization during the early phase of the COVID-19 pandemic: viral fear or lockdown effect? *Disaster Med Public Health Prep*. 2022;16(1):36–9. 32782063 10.1017/dmp.2020.303 PMC7503047

[48] Centers for Disease Control and Prevention. PLACES Data [online]. *National center for chronic disease prevention and health promotion, division of population health*. 2022. https://www.cdc.gov/PLACES. Accessed 11 May 2023.

[49] Yland JJ, Wesselink AK, Lash TL, Fox MP. Misconceptions about the direction of bias from nondifferential misclassification. *Am J Epidemiol*. 2022;191(8):1485–95. 35231925 10.1093/aje/kwac035 PMC9989338

[50] D’Adamo A, Schnake-Mahl A, Mullachery PH, Lazo M, Roux AVD, Bilal U. Health disparities in past influenza pandemics: a scoping review of the literature. *SSM-Population Health*. 2022;21:101314. 36514788 10.1016/j.ssmph.2022.101314 PMC9733119

