# Supplementary material for: Area-Level Social Vulnerability and Severe COVID-19: A Case–Control Study Using Electronic Health Records from Multiple Health Systems in the Southeastern Pennsylvania Region
Source: J Urban Health. 2024 May 13;101(4):845–55. doi: 10.1007/s11524-024-00876-6 (PMC11329477; doi:10.1007/s11524-024-00876-6)
Supplement: Supplementary file 1 — Supplementary file1 (DOCX 4012 KB) [file 11524_2024_876_MOESM1_ESM.docx]

**Supplementary Figure 1: Population distribution from census data vs. distribution of patients from the HSX clinical data repository**


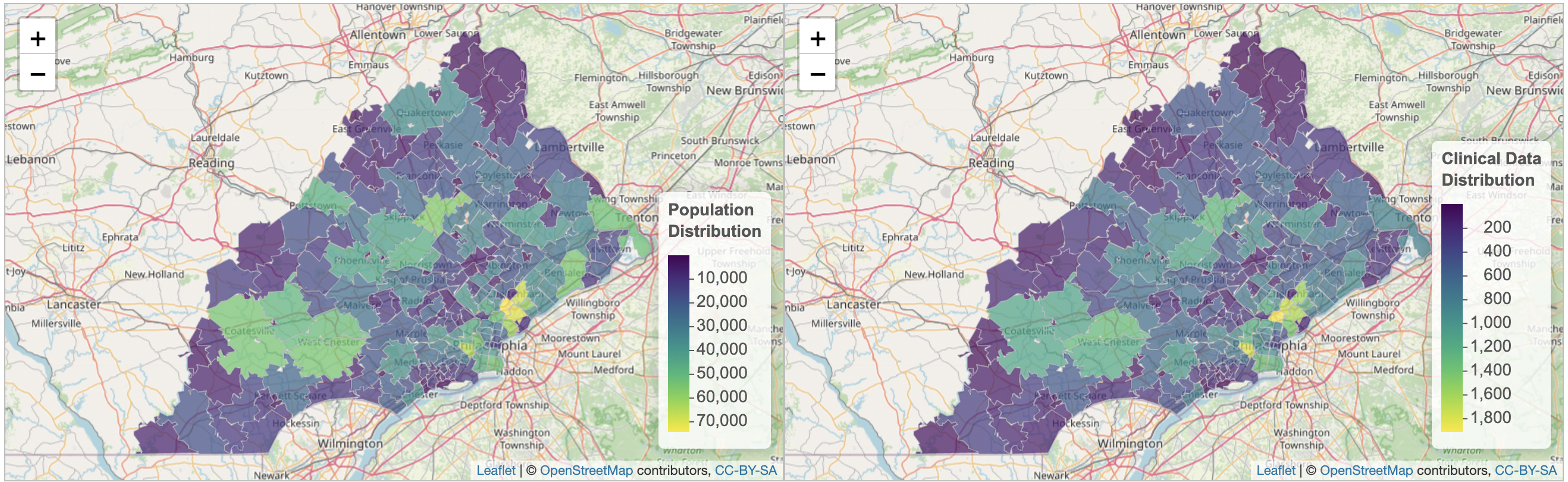


**Supplemental Box 1: List of ICD-10 codes for chronic conditions/comorbidities.**

Diabetes codes: E10 E11 E12 E13

Heart condition codes: I05 I06 I07 I08 I09 I22 I25 I27 I28 I31 I34 I35 I36 I37 I38 I42 I44 I45 I70 I71 I72 I77 I78 I79 I20.9 I51.0 I51.1 I51.2 I51.4 I51.5 I51.6 I51.7 I51.8 I48.2 I95.8

Renal disease codes: N18 N19

Liver disease codes: K72.1 E83.0 K70 K71 K73 K74 K75 K76

Immunocompromised state codes: D80 D81 D82 D83 D84 D86 D89 C94.4 D61.81 D72.81 D76 T86 Z48.2 Z49 Z94 B20 B59 C80.2 C88.8 C94.6 D46.22 D47.1 D47.9 D47.Z1 D46.22 D47.1 D47.9 D47.Z1 D47.Z9 D61.09 D70.0 D70.1 D70.2 D70.4 D70.8 D70.9 D71 D72.0 D73.81 D75.81 D75.81 E40 E41 E42 E43 I12.0 I13.11 I13.2 K91.2 M35.9 N18.5 N18.6 Z99.2

Hypertension codes: I10 I11 I12 I13 I15

Cancer codes: C
